# Multilevel analysis of determinants of polygyny among married men in Ethiopia

**DOI:** 10.1186/s12889-021-11701-z

**Published:** 2021-09-15

**Authors:** Yitayish Damtie, Bereket Kefale, Melaku Yalew, Mastewal Arefaynie, Bezawit Adane

**Affiliations:** 1 Department of Reproductive and Family Health, School of Public Health, College of Medicine and Health Sciences, Wollo University, PO Box: 1145, Dessie, Ethiopia; 2 Department of Epidemiology and Biostatistics, School of Public Health, College of Medicine and Health Sciences, Wollo University, Dessie, Ethiopia

**Keywords:** Polygyny, Polygamy, Married men, Marriage, Ethiopia

## Abstract

**Background:**

Polygyny occurs when a man has more than one wife at the same time. It often contributes to poor health among family members, particularly young children. It encourages the spread of sexually transmitted infections (STIs) including HIV/AIDS. The determinants of polygyny have not yet been adequately explored in Ethiopia. This study adds to the body of knowledge concerning the prevalence and distribution of polygyny in the country.

**Methods:**

This study is a secondary analysis of the 2016 Ethiopian Demographic and Health Survey (EDHS) data. Using a two-stage stratified cluster sampling, 7470 married men were selected. After verifying the assumptions of multilevel logistic regression analysis, Stata version 14.0 was used to analyse the data. A multilevel mixed-effects logistic regression model was used to identify predictors of polygyny. An adjusted odds ratio with a 95% confidence interval was used to measure the association. A *p*-value of < 0.05 was considered to indicate statistical significance.

**Results:**

Age from 30 to 44 years [AOR = 5.78, 95% CI = (3.13, 10.7)], age from 45 to 59 years [AOR = 16.5, 95% CI = (8.59, 31.8)], men with primary education or no formal education [AOR = 3.40, 95% CI = (1.50, 7.69)], being Muslim [AOR = 2.47, 95% CI = (1.28, 4.77)], sexual initiation at or above the age of 18 years [AOR = 0.46, 95% CI = (0.30, 0.68)] and being from a less developed region of Ethiopia [AOR = 3.67, 95% CI = (2.30, 5.83)] were factors associated with polygyny.

**Conclusion:**

Both individual and community level factors were identified as predictors of polygyny. Improving educational attainment and delaying men’s sexual debut could encourage the reduction of polygyny in Ethiopia.

## Background

Polygamy is a form of marriage involving multiple spouses. It may occur as polygyny (when a man has multiple wives concurrently, and less commonly, as polyandry (when a woman has multiple husbands concurrently) and as polygynandry (concurrent marriage of two or more wives to two or more husbands) [1–4].

Polygyny is the commonest form of polygamy. It existed historically in more than 80% of preindustrial societies [5]. Although the global prevalence of polygyny is small, more than a third of the world’s population lives in a community that permits it [6]. The history of polygyny has been practiced for many centuries by various cultures in the world. It has existed as a fundamental part of family law in most African countries. With the arrival of Christianity and colonists, however, it came to be considered as a form of slavery that needed to be eliminated. As a result, its prevalence has been diminishing for decades. Nevertheless, it remains more common in Sub-Saharan Africa (SSA) than anywhere else [7]. The highest prevalence of polygyny in Africa is found across the so-called ‘polygyny belt’ stretching from Senegal in West Africa to Tanzania in East Africa [8]. As the Demographic and Health Survey (DHS) data show, 11, 27, and 53% of marriages in Zimbabwe, Ivory Coast, and Guinea were polygynous respectively [9]. Another DHS reported that polygyny represents 25% of all marriages in the Democratic Republic of Congo (DRC), 47% in Sierra Leone, and 53% in The Gambia [10].

In Ethiopia, although polygyny has declined in recent decades, the national average of polygyny among currently married men was 11% in 2016 and ranged from 1% in the Amhara region to 29% in the Somali region [11, 12].

There are several possible reasons for the persistence of polygyny in Ethiopia. Demographic factors such as high infant and child mortality, high male mortality and out-migration, and potentially lethal activities performed by men such as hunting and military combat resulting in a surplus of women and shortage of men, all of which can encourage polygyny [13, 14]. Religions, mainly Mormonism and Islam, have increased the prevalence of polygyny. Polygyny among Mormons is encouraged whereas, in Islam, it is merely permitted [13, 15]. The prevalence of polygyny is also affected by age, place of residence, and household wealth [16, 17]. Culture and tradition are the other main factors contributing to the acceptance of polygyny. Polygyny in many African cultures is considered to be a solution for women’s infertility and menopause. It also allows men to satisfy sexual needs while their wives are pregnant since sexual relations during pregnancy are taboo in some cultures. Some believe that it is important for a man to continue his family name in future generations. In agricultural societies, polygyny leads to more children who can work in housework, farming, and cattle herding [4, 7, 18, 19].

Polygyny is legally prohibited in Ethiopia by the revised family code proclamation since it undermines women’s self-worth, violates gender equality and women’s rights, both of which are protected under the Convention on the Elimination of All Forms of Discrimination Against Women (CEDAW) and the Universal Declaration of Human Rights (UDHR) [20]. These legal restrictions on polygyny, however, are rarely enforced.

In addition, polygyny increases the spread of HIV/AIDS and other Sexually Transmitted Diseases (STDs) [21–23]. Men in a polygynous marriage were 2.6 times more likely to be HIV positive than monogamous men [24] and 2.9 times more likely to be infected with Herpes Simplex Virus (HSV)-2 [25]. Moreover, polygyny is associated with a higher incidence of infant and child mortality [26, 27]. Children from polygynous families experience ill health and early childhood death as a result of malnutrition and HIV/AIDS [28–30]. Adolescents in polygynous families are more likely to have lower self-esteem [31] and poor academic outcomes compared to adolescents from monogamous families [2, 32–34]. Polygyny is also a source of emotional distress and depression imposing detrimental effects on the mental health of wives [35–37].

Identifying the determinants of polygyny is important to meet Sustainable Development Goal (SDG) 3 (to end the HIV epidemic and to decrease neonatal mortality below 12 per 1000 live births by 2030), SDG 5 (to achieve gender equality and empower all women and girls by 2030) [38] and Ethiopian Health Sector Transformation Plan (HSTP) IV (to decrease the under-five mortality rate from 64 to 30 per 1000 live births, to reduce adult HIV incidence by 60% below 2010 incidence, and to achieve zero new HIV infections among children by 2020) [39].

The reasons for polygyny are multifaceted and vary within and across countries. However, the polygyny practice is more prevalent in sub-Saharan African countries than other world sub-regions [40]. The problems associated with polygyny are numerous, especially in a less developed country like Ethiopia where there is poverty and majority of families earn less than $1 per day [41]. Polygyny has been implicated in many studies as one of the factors that promote early marriage, domestic violence, harmful traditional practices, and high fertility [42–45]. Despite the public health importance of polygyny, it is often less studied in Ethiopia. This study aimed to determine individual and community-level factors associated with polygyny among married men in Ethiopia.

## Methods

### Study setting

This study was conducted in Ethiopia, a country with more than 80 ethnic groups [46]. According to the census conducted in 2007, the largest ethnic groups in Ethiopia are Oromo, Amhara, Somali, and Tigray which constituted 43.4, 26.9, 6.2, and 6.1% of the country’s total population respectively [47]. Each ethnic group has a distinct culture, set of customs, traditions, and languages specific to their ethnicity. There are 87 native languages spoken in Ethiopia. They can be classified into Semitic, Cushitic, Omotic, and Nilotic groups. Of these, Amharic is the most common working language while English is the second language of the federal government [46, 48]. The country also has a long-standing connection with Christianity (Catholic, Orthodox, and Protestant), Islam, and less commonly with Judaism and Paganism [48]. The country’s two largest religious faiths are Orthodox Christianity (43.5%) and Islam (33.9%) [47].

### Data source and study population

This study is a secondary analysis of data from the most recent (2016) EDHS conducted by the central statistical agency with the collaboration of Ethiopian Public Health Institute, Federal Ministry of Health, and the Inner City Fund International which provides technical assistance through its MEASURE DHS project; a USAID-funded program supporting the implementation of population and health surveys in countries worldwide. All of the 7470 married men interviewed in the 2016 EDHS were included in the study.

### Conceptual framework and variable measurement

**Fig. 1:**
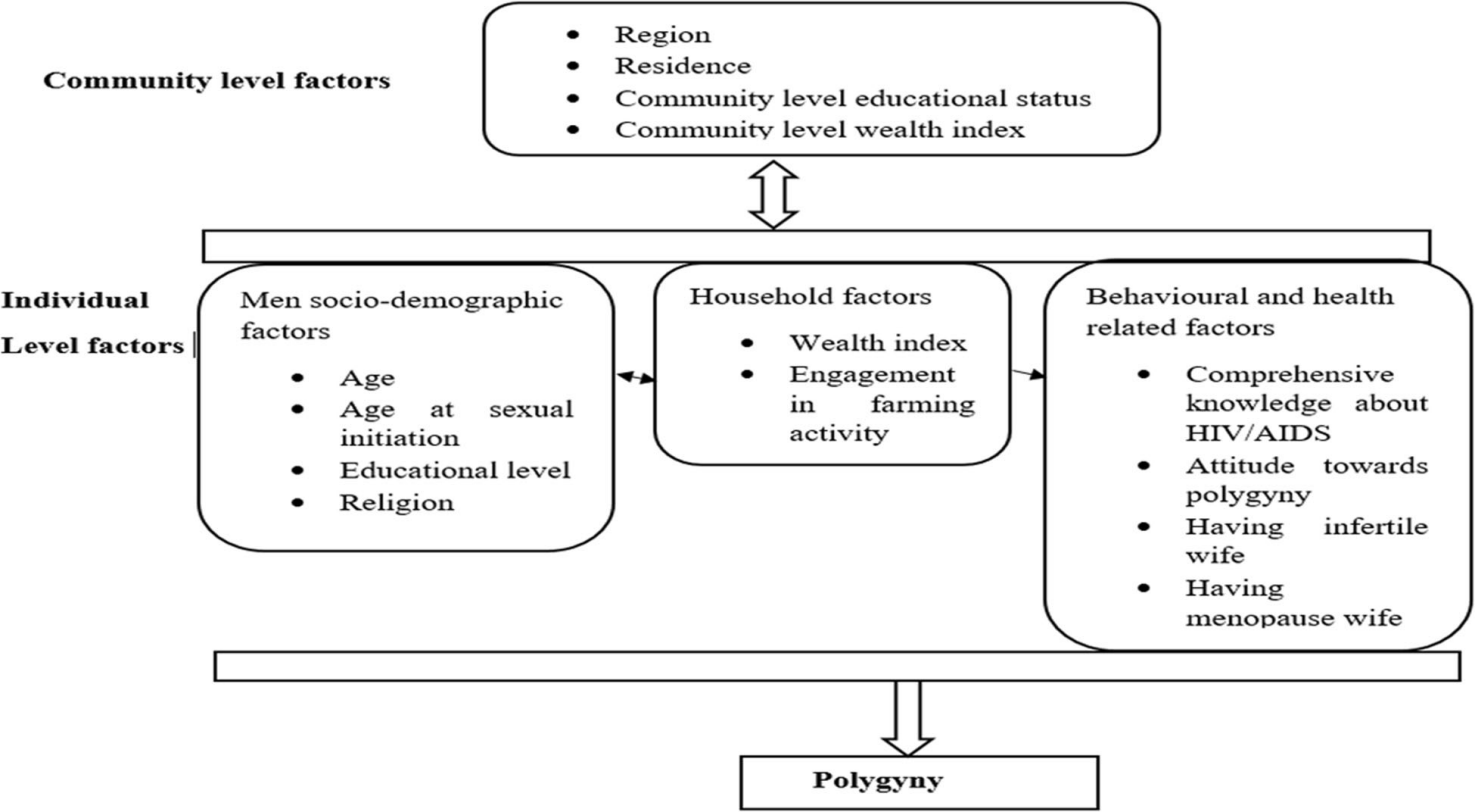
Conceptual framework showing factors associated with polygyny among married men in Ethiopia, EDHS 2016 [6, 13–19, 40, 49–52]

The dependent variable (polygyny) was dichotomized as “Yes/No.” A marriage is said to be polygynous if a man has more than one wife at the time of the interview [49]. The independent variables were both individual and community-level factors. Individual-level factors include socio-demographic factors, household factors and behavioural and health-related factors. Community-level factors were region, place of residence, community-level wealth status and community-level educational status (Fig. 1).

Regions were categorized as less developed (Afar, Somali, Benishangul Gumuz, and Gambela) or more developed (Addis Ababa, Dire Dawa, Amhara, Tigray, Oromia, Southern Nation Nationalities and Peoples (SSNP) region and Harari) [53]. In addition, a total of two community-level factors were generated by aggregating men’s characteristics in every 645 clusters. In the 2016 EDHS, a two-stage stratified sampling technique was used for sample selection during the data collection exercise. In the first, a total of 21 sampling strata’s were created from each regions after stratifying them into urban and rural areas. Then a total of 645 (202 in urban areas and 443 in rural areas) non-overlapping smaller geographical units (tagged as clusters) were selected. In the second stage, 28 households per cluster were selected and included in the survey.

**Fig. 2:**
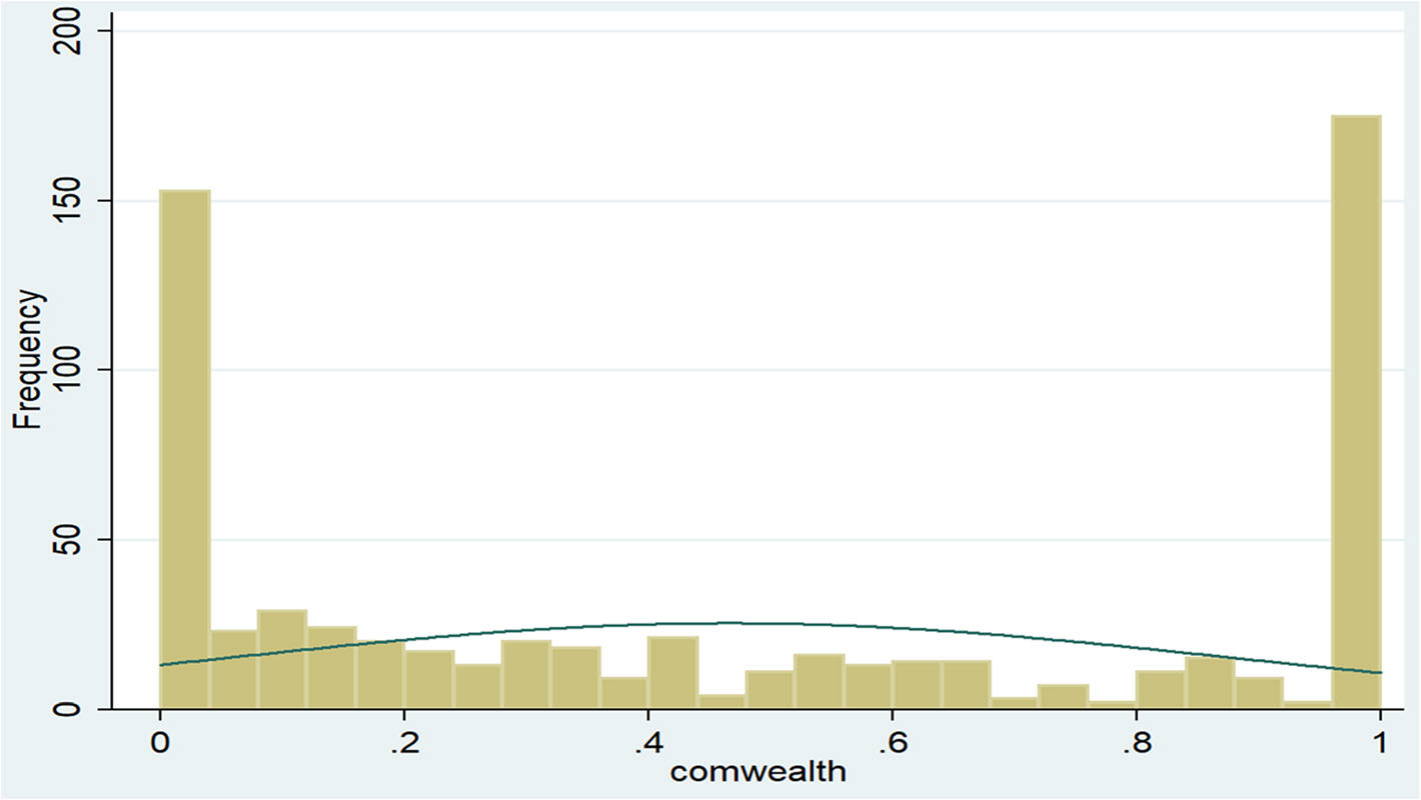
The histogram used to check the normality of the distribution of community-level wealth status in Ethiopia, EDHS 2016

Community-level wealth status was generated by dividing the percentage of men with a wealth score of less than 33.3% (poor wealth index) to the total wealth index in every 645 clusters. As the Shapiro–Wilk normality test (Table 1) and the histogram (Fig. 2) showed, the community-level wealth status was not normally distributed (*p* = 0.00000) and the median value was used as a cut-off point to categorize community wealth status.

**Table 1:** Shapiro-Wilk test to check the normality of the distribution of community-level wealth status in Ethiopia, EDHS 2016

Variable	Observations (N)	Shapiro–Wilk test statistics (W)	index for departure (V)	z- score	Prob>z (*p*-value)
Community level wealth status	645	0.95648	18.376	7.076	0.00000

**Fig. 3:**
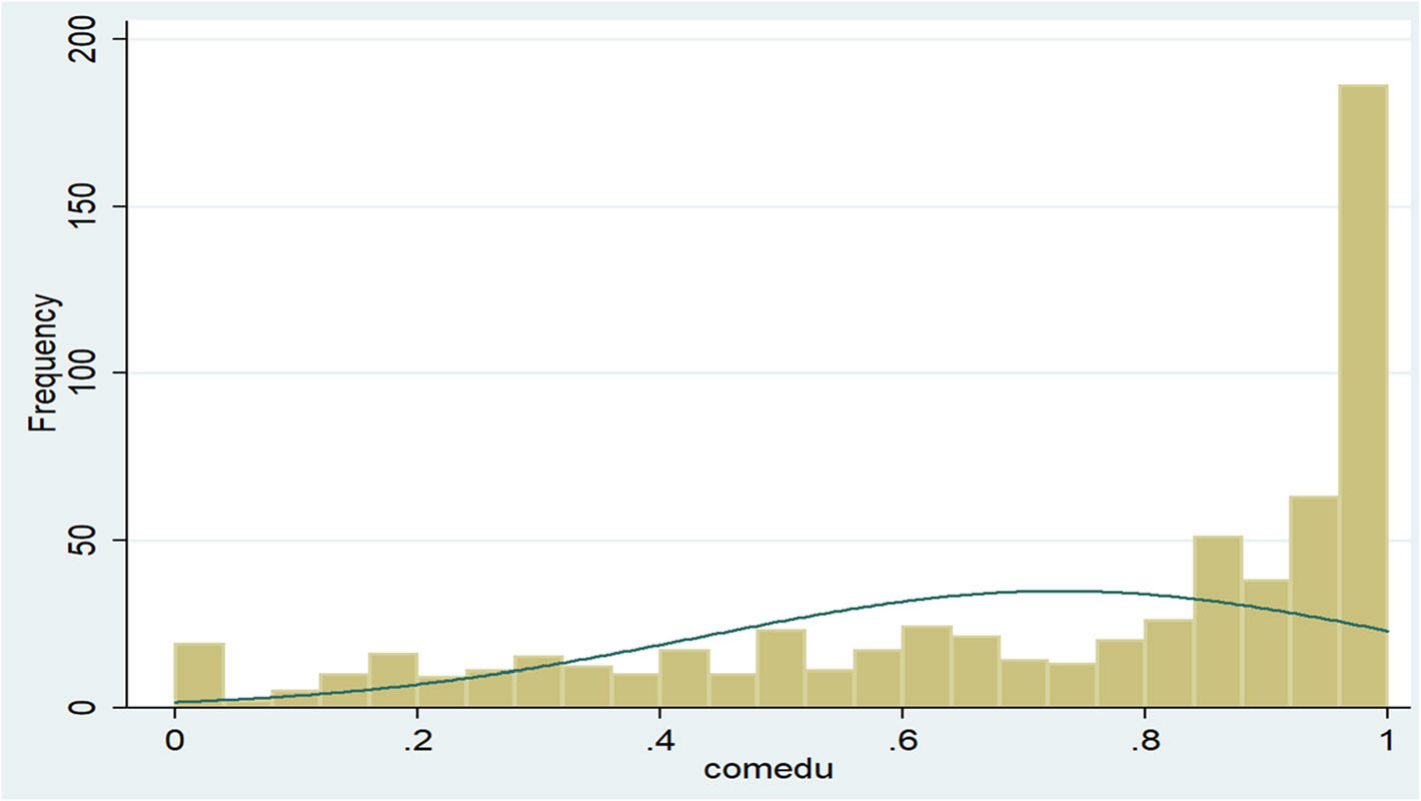
The histogram used to check the normality of the distribution of community-level educational status in Ethiopia, EDHS 2016

Similarly, community-level educational status was computed by using the percentage of men with the two lowest levels of educational attainment (no education and primary education) in each 645 non-overlapping smaller geographical units tagged as clusters. The distribution of community-level educational status was checked by using the Shapiro–Wilk normality test (Table 2) and the histogram (Fig. 3). Since the data was not normally distributed (*P* = 0.00000), the median value was used to categorize community-level educational status.

**Table 2:** Shapiro-Wilk test to check the normality of the distribution of community-level educational status in Ethiopia, EDHS 2016

Variable	Observations (N)	Shapiro–Wilk test statistics (W)	index for departure (V)	z- score	Prob>z (*p*-value)
Community-level educational status	645	0.92592	31.280	8.369	0.00000

### Data processing and analysis

The data were cleaned, re-categorized, and analysed by Stata/SE version 14.0. Descriptive statistics such as frequencies, percentages, and medians were computed and the results were presented in tables and texts. Sample weight was used for the compensation of non-responses and unequal selection of samples between each stratum. The Intra-class Correlation Coefficient (ICC) for this study was 38.1%. Due to this and hierarchical nature of EDHS data, a multi-level mixed-effects logistic regression model was used to determine individual and community level factors associated with polygyny. Multi-level mixed-effect analysis is an analytical strategy allowing the simultaneous examination of individual-level and group-level factors. It recognizes group effects and hierarchical structures leading to correct conclusion. On the other hand, individual-level analysis treat independent observations as the units of analysis. It fails to recognize the group effect leading to underestimation of the standard errors of the regression coefficients and overstatement of statistical significance.

The log of the likelihood of being in a polygynous marriage can be modelled as follows [54];

$$ \mathrm{Log}\left[\frac{\pi ij}{1-\pi ij}\right]={\upbeta}_0+{\upbeta}_1{\mathrm{X}}_{\mathrm{ij}}+{\mathrm{B}}_2{\mathrm{Z}}_{\mathrm{ij}}+{\upmu}_{\mathrm{j}}+{\mathrm{e}}_{\mathrm{ij}}. $$

Where,

- i is the individual unit and j is the community units.
- πij is the likelihood of being in a polygynous marriage for the i^th^ man in the j^th^ community.
- 1 − πij is the odds of being in a monogamous marriage.
- X and Z represent individual and group-level factors respectively.
- The intercept (β0) represents the effect on the likelihood of being in a polygynous marriage in the absence of the effect of predictor variables.
- β’s are the fixed coefficients.
- µj shows the effect of community level factors on polygyny for the j^th^ community i.e. the random effect.
- eij indicates the random errors occurred at the individual levels.

Both bi-variable and multivariable multilevel logistic regression analyses were estimated and a *p*-value of < 0.25 was used to screen eligible variables for multivariable multilevel logistic regression analysis. Four models were fitted: Model 0 also known as an empty model (without any predictor variable); Model 1 (include only individual-level predictors); Model 2 (include only group-level predictors) and Model 3 (include both individual and group-level factors). AOR with a 95% CI was used to measure the association between polygyny and various predictor variables (fixed-effects). The presence of statistical significant association was declared at a *p*-value < 0.05.

The variation between each cluster was reported using Proportional Change in Variance (PCV), ICC and Median Odds Ratio (MOR). The ICC was used to show the degree in which the observation within one cluster resembled each other. It was calculated by using the following formula: [ICC= $\frac{\ {\updelta}^2}{\ {\updelta}^{2+\frac{\uppi^2}{3}}}$], where δ^2^ is the cluster variance.

MOR is the odds ratio between the area at the lowest risk and at the highest risk when two areas are peaked arbitrarily. It was calculated as follows: [MOR = exp. ($\sqrt{2{\mathrm{x}\updelta}^2+0.6745}$) ≈ exp(0.95δ)]. PCV indicates the total disparity attributed by individual and area-level factors in the multilevel mixed effect model. Log-likelihood test and standard error at the cut-off point of _±_2 were used to check model fitness and multicollinearity respectively.

## Results

### Characteristics of the respondents

Among the 7470 married men included in the analysis, 3811 (51%) were 30–44 years old, 1254 (16.8%) completed secondary and post-secondary education, 3182 (42.6%) men were Orthodox Tewahido, and 2873 (38.5%) men were poor (having a wealth score of less than 33%). Among study participants, 1393 (18.6%) had their first sexual encounter before age 18 years, 1189 (15.9%) married men resided in rural areas and 7115 (95.2%) men were from more developed regions. Among all respondents, 4801(64.3%) men had no comprehensive knowledge of HIV/AIDS and 3788 (50.7%) were from communities with a high proportion of poor men (Table 3).

**Table 3:** Individual and community-level characteristics of married men in Ethiopia, EDHS 2016 (*n* = 7470)

Variable	Category	Number of respondents	Percentage (%)
Age	15–29 years	1727	23.1
	30–44 years	3811	51.0
	45–59 years	1932	25.9
Education	Primary and below	6216	83.2
	Secondary and above	1254	16.8
Wealth index	Poor	2873	38.5
	Middle	1501.	20.1
	Rich	3096	41.4
Religion	Orthodox Tewahido	3182	42.6
	Muslim	2522	33.8
	Protestant	1612	21.6
	Others^a^	154	2
Age at sexual initiation	< 18 years	1393	18.6
	≥18 years	6077	81.4
Comprehensive HIV knowledge	Knowledgeable	4801	64.3
	Not knowledgeable	2669	35.7
Residence	Urban	1189	15.9
	Rural	6281	84.1
Region	More developed	7115	95.2
	Less developed	355	4.8
Community educational status	Low	3120	41.8
	High	4350	58.2
Community wealth status	Low	3682	49.3
	High	3788	50.7

^a^ Catholic and traditional religion follower

### Individual and community-level factors associated with polygyny

In the final model (model 3) age, educational status, religion, and age at sexual initiation from individual-level factors and region from community-level factors had a statistically significant association with polygyny. Study participants aged from 30 to 44 years were 5.78 times more likely to practice polygyny compared to married men aged 15–29 years [AOR = 5.78, 95% CI = (3.13, 10.7)]. Similarly, married men aged 45–59 years were 16.5 times more likely to practice polygyny compared to married men aged 15–29 years [AOR = 16.5, 95% CI = (8.59, 31.8)].

Uneducated men and men with only primary level education were 3.4 more likely to practice polygyny than married men who completed secondary and post-secondary education [AOR = 3.40, 95% CI = (1.50, 7.69)]. Muslim married men were 2.47 times more likely to practice polygyny than Orthodox Tewahido men [AOR = 2.47, 95% CI = (1.28, 4.77)].

Married men who initiated sex at or above the age of 18 years were 54% less likely to practice polygyny compared to married men who initiated sex under the age of 18 years [AOR = 0.46, 95% CI = (0.30, 0.68)]. Men in less developed regions of Ethiopia were 3.67 more likely to practice polygyny compared to men in more developed regions [AOR = 3.67, 95% CI = (2.30, 5.83)] (Table 4).

**Table 4:** Multilevel logistic regression analysis of individual and community-level factors associated with polygyny among married men in Ethiopia, EDHS 2016 (*n* = 7470)

Individual and community-level factors	COR (95% CI)	Model 0 ICC = 38.1%	Model 1 AOR (95% CI)	Model 2 AOR(95% CI)	Model 3 AOR (95% CI)
Age					
15–29 years	1		1		1
30–44 years	5.60 (3.10,10.1)		5.73 (3.1, 10.6)		**5.78 (3.13, 10.7)**
45–59 years	16.0 (8.69,29.6)		16.1 (8.37, 30.9)		**16.5 (8.59, 31.8)**
Education					
Primary and below	5.26 (2.61,10.6)		3.87 (1.83, 8.20)		**3.40 (1.50, 7.69)**
Secondary and above	1		1		1
Religion					
Orthodox	1		1		1
Muslim	3.33 (1.92,5.79)		3.57 (1.93, 6.62)		**2.47 (1.28, 4.77)**
Protestant	1.98 (0.96,4.07)		2.23 (1.03, 4.82)		1.97 (0.90, 4.30)
Other^a^	2.98 (0.76,11.7)		3.48 (0.88, 13.7)		2.98 (0.77, 11.6)
Age at sexual initiation					
under 18 years	1		1		1
At or above 18 years	0.54 (0.38,0.77)		0.45 (0.30, 0.67)		**0.46 (0.30, 0.68)**
Comprehensive knowledge of HIV/AIDS					
Knowledgeable	1.31 (0.89,1.93)		1.20 (0.82, 1.76)		1.12 (0.76, 1.66)
Not knowledgeable	1		1		1
Residence					
Urban	1			1	1
Rural	4.96 (2.51,9.79)			4.42 (2.10, 9.28)	3.00 (0.32, 6.82)
Region					
More developed	1			1	1
Less developed	4.63 (3.26,6.58)			4.36 (2.99, 6.35)	**3.67 (2.30, 5.83)**
Community-level educational status					
Low	1			1	1
High	1.51 (1.00,2.28)			0.92 (0.60, 1.41)	0.79 (0.49, 1.26)
Community-level wealth status					
Low	1			1	1
High	0.43 (0.29,0.65)			0.87 (0.54, 1.39)	0.94 (0.57, 1.53)

Model 0 (null model) - without independent predictors, Model 1- only individual-level factors, Model 2- only community-level factors; Model 3- both individual and community-level factors; ^a^ Catholic and traditional religion follower; *COR* crude odds ratio, *AOR* adjusted odds ratio

### Random effects (measures of variation)

The practice of polygyny significantly varies across 645 non-overlapping smaller units tagged as clusters. ICC indicated, 38.1% of the variation in practicing polygyny was attributed to community-level factors. PCV in the final model showed 10.3% of the variation in polygyny across communities was explained. Likewise, MOR for polygyny in the null model was 3.87 which shows the presence of variation across each cluster (Table 5).

**Table 5:** Measure of variation for polygyny at cluster level in multilevel logistic regression analysis, EDHS 2016

Measure of variation	Model 0 (95% CI)	Model 1 (95% CI)	Model 2 (95%CI)	Model 3 (95%CI)
Variance	2.03 (1.56, 2.62)	1.93 (1.40, 2.67)	1.84 (1.40, 2.40)	1.82 (1.39, 2.71)
ICC (%)	38.1	36.9	35.9	35.6
PCV (%)	Reference	4.9	9.4	10.3
MOR	3.87	3.74	3.63	3.60
Model fitness				
Log-likelihood	− 1512.2	− 1347.5	− 1481.2	− 1331.4

Model 0 (null model)- without independent predictors, Model 1- only individual-level factors, Model 2- only community-level factors, Model 3- both individual and community-level factors, ICC-Intra-class correlation coefficient, PCV- Proportional change in variance, and MOR- Median odds ratio

## Discussion

Men between the ages of 30 and 59 years were more likely to be in a polygynous marriage than younger age men. The reason could be men aged 30 and older are more likely to be non-educated and lead their lives based on cultural traditions. In addition, men over 30 years are more likely to be economically stable and so able to afford multiple wives [55, 56].

Being Muslim was positively associated with polygyny in Ethiopia. That was also found in a study in SSA and Ghana [40, 49] and in a cross-national analysis of 26 African countries [57]. The reason could be polygyny’s encouragement by Islam. Islamic doctrine permits a man to have up to four wives for creating and sustaining the Muslim family and to populate the world with believers [58].

Being uneducated or educated only at the primary level was positively associated with polygyny. That was also found in a study conducted in the city of Bastak, Iran [51]. Polygyny is a marriage of a man with several women which is a common practice in Africa including Ethiopia. The practice is more prevalent among poorly educated men or those with no formal education than their highly educated counterparts. Education provides information that can change the orientation of men towards this social practice and previous studies have established that good education influences small family size. Many men in Africa are in polygyny marriage today because of their interest in large family size which in some cases is a cultural demand in traditional African settings [40].

Initiating sex before age 18 is positively associated with polygyny. That was also found in a study in Uganda [52]. The possible justification could be, individuals who initiated sex before age 18 are more likely to adapt and maintain the behavior for a long time and will have multiple, casual, and concurrent sexual partnerships at a later age [59].

Living in less developed regions within Ethiopia also has a positive association with polygyny similar to a study conducted in Ghana, Senegal, Kenya, and Zimbabwe [40]. The possible reason is that most men in less developed regions are agrarian as a result, they tend to marry more than one wife and produce several children to ensure division of labor and enhance productivity. Moreover, people in less developed regions are dogma-bound and affected by the deep-rooted traditional beliefs and values which makes them less penetrated by modernization. On the other hand, people in urban settings are highly affected by western lifestyle and have a chance to media exposure so that traditional polygamy is no longer the norm and are likely to practice monogamous marriage [7, 19, 50].

## Summary

Even though polygynous marriage is formally prohibited by the revised family code proclamation [20], it is quite common in Ethiopia where 11% of currently married men practice polygynous marriage [11]. Polygynous marriage adversely affects the health of the child and wives. It also violates gender equality and women’s rights which are protected under different conventions. Improving maternal and child health, achieving gender equality, and ending HIV/AIDS epidemic are the primary intervention areas of the government that have to be achieved by 2030 [38]. So, the national government should synthesize legal institutions to enforce legal restrictions according to the proclamation. In addition, efforts should be made to delay sexual debut and to increase the education service coverage among men in all regions of the nation.

This study has strengths and limitations. By considering the variation among 643 non-overlapping smaller geographical units (clusters) a multilevel mixed-effects logistic regression model was used to identify individual and community-level factors. Furthermore, the findings of this research can be generalized to the Ethiopian population since the study was done on nationally representative data. A limitation of this study is its lack of data on infertility and menopause of the first wife and occupation, most especially farming. The inability to determine causality is inevitable due to the cross-sectional nature of the data.

## Conclusions

Individual-level factors; age, religion, educational status, and age at sexual initiation and the community-level factor region had statistically significant associations with polygyny. So, the preexisting strategies and policies about universal education to all should be the area of emphasis especially for those who are uneducated or educated only at the primary level. Furthermore, extra efforts are still required to change the values and attitudes of the people towards polygyny. Sexuality education should also be given to delay age at sexual initiation and the government should give special attention to less developed regions. Further qualitative research to explore different beliefs, customs, cultures, and traditions associated with polygyny is recommended. Further research using spatial analysis is also recommended to provide area-specific interventions.

## Data Availability

The datasets used and/or analysed during this study are available from the corresponding author on reasonable request.

## Abbreviations

AOR: Adjusted Odds Ratio
CI: Confidence Interval
CEDAW: Convention on the Elimination of All Forms of Discrimination against Women
DRC: Democratic Republic Congo
EDHS: Ethiopia Demographic and Health Survey
HSTP: Health Sector Transformation Plan
HSV: Herpes Simplex Virus
HIV/ AIDS: Human Immunodeficiency Virus/ Acquired Immune Deficiency Syndrome
ICC: Intra-cluster Correlation Coefficient
MOR: Median Odds Ratio
PCV: Proportional Change in Variance
STD: Sexual Transmitted Diseases
SNNP: Southern Nation Nationalities and Peoples
SSA: Sub Saharan Africa
SDG: Sustainable Development Goals
UDHR: Universal Declaration of Human Rights
